# Melatonin for Neuropathic Pain: Protocol for a Double-blind, Randomized Controlled Trial

**DOI:** 10.2196/40025

**Published:** 2022-09-28

**Authors:** Ian Gilron, Dongsheng Tu, Ronald R Holden, Dwight E Moulin, Scott Duggan, Roumen Milev

**Affiliations:** 1 Department of Anesthesiology & Perioperative Medicine Queen's University Kingston, ON Canada; 2 Department of Psychology Queen's University Kingston, ON Canada; 3 Western University London, ON Canada

**Keywords:** melatonin, neuropathic pain, chronic pain, sleep, analgesic therapy, placebo, clinical trials, neuropathic, pain, nonopioid, treatment, efficacy, insomnia, placebo, preclinical, clinical

## Abstract

**Background:**

Neuropathic pain (NP), a complication of several conditions (eg, diabetic neuropathy and varicella zoster), is a common challenging problem, and there is a growing need to develop safe and effective nonopioid treatments. Sleep disturbance is commonly associated with NP because pain intensity in NP conditions is often worse at night. The pineal hormone melatonin has been shown to reduce pain in both preclinical and clinical settings, in addition to multiple trials demonstrating efficacy for primary insomnia and delayed sleep phase syndrome.

**Objective:**

We propose to conduct a clinical trial to evaluate the efficacy and safety of melatonin for NP.

**Methods:**

Using a double-blind, placebo-controlled, crossover design, 30 adults with NP will be randomly allocated to one of two sequences of treatment with melatonin and placebo. During each of the two treatment periods, participants will take capsules containing melatonin or placebo for 4 weeks, followed by a 7-day washout period. The primary outcome will be mean daily pain intensity (scored 0-10) at maximally tolerated doses (MTDs) during each period. Secondary outcomes, assessed at MTDs, will include global improvement, adverse events, mood, and quality of life.

**Results:**

This trial was registered in the International Standard Randomized Controlled Trial registry May 4, 2022 (ISRCTN #16215617), attained conditional ethics approval May 9, 2022 (Queen’s University Health Sciences & Affiliated Teaching Hospitals Research Ethics Board protocol number ANAE-387-22), and recruitment is set to start August 2022.

**Conclusions:**

This trial will provide rigorous evidence comparing the efficacy of melatonin to that of placebo in the treatment of NP.

**Trial Registration:**

ISRCTN Registry 16215617; https://www.isrctn.com/ISRCTN16215617

**International Registered Report Identifier (IRRID):**

PRR1-10.2196/40025

## Introduction

Neuropathic pain (NP) is a common form of secondary chronic pain due to a lesion or disease of the nervous system [1] such as diabetic neuropathy, spinal radiculopathy, HIV-neuropathy, postherpetic neuralgia, and cancer-related conditions [2]. NP has been estimated to have a prevalence of 7%-8% [3] and is known to impair physical, social, and occupational function with a subsequent devastating impact on patients, their families, and society [4,5]. Oral medications may be a valuable element of multimodal NP management owing to their ease of administration and engagement of drug effect sites throughout the sensory nervous system [6,7]. However, studied treatments provide only partial benefit owing to incomplete efficacy and dose-limiting adverse effects (AEs) [8].

Chronic pain, including NP conditions, is commonly associated with sleep disturbance. Complex interactions between pain and sleep are such that effective therapy requires coordinated attention to the interaction between pain and sleep [9,10]. In patients with chronic pain, observations suggest that a night of poorer sleep is followed by a more painful day, and a more painful day is followed by a night of poorer sleep [11]. Thus, in addition to treating one of the most prominent secondary features of chronic pain, pain therapies that also improve sleep may be expected to have more favorable efficacy [12].

Melatonin (N-acetyl-5-methoxytryptamine), a hormone secreted by the pineal gland, has been implicated in several homeostatic functions including sleep, modulation of circadian rhythms and mood, enhanced immunity, and antioxidant free radical scavenging [13]. Relevant to pain, accumulating preclinical and clinical evidence suggests antinociceptive effects of melatonin by acting on MT_1_ and MT_2_ membrane receptors (to reduce cyclic AMP) but possibly also (1) by indirectly activating opioid receptors, (2) by inhibiting the production of proinflammatory cytokines, (3) by activating γ-aminobutyric acid-A receptors, and (4) through its antioxidant effects [14]. Emerging evidence suggests that melatonin reduces pain in both preclinical and clinical (fibromyalgia) settings [15-17], in addition to multiple randomized controlled trials (RCTs) demonstrating efficacy for primary insomnia and delayed sleep phase syndrome [18]. After oral administration, melatonin undergoes first-pass hepatic metabolism [19], with subsequent secondary renal metabolism and elimination [20]. A review of studies involving varying doses [21] reported that oral administration of melatonin results in a transport maximum ranging 15-210 minutes, and a half-life ranging 28-126 minutes [21].

Thus, in the interest of further evaluating the analgesic efficacy of melatonin for NP, our objective is to conduct a double-blind RCT to compare the efficacy of melatonin to that of placebo in patients with chronic neuropathic conditions.

## Methods

### Ethics Approval

This study has been submitted to, and is currently under review by, the Queen’s University Health Sciences & Affiliated Teaching Hospitals Research Ethics Board (protocol #ANAE-387-22). This study protocol will be conducted in accordance with the principles of the Declaration of Helsinki, Initiative on Methods, Measurement, and Pain Assessment in Clinical Trials’ guidelines [22,23], and in accordance with the International Council for Harmonisation Good Clinical Practice: Consolidated guideline. This trial has been funded by the Physicians’ Services Incorporated Foundation and was registered in the International Standard Randomized Controlled Trial Registry on May 4, 2022 (ISRCTN #16215617).

### Aims and Hypothesis

The objective of this trial is to evaluate the efficacy, safety, and tolerability of melatonin in treating pain in participants with chronic neuropathic conditions. Our primary hypothesis is that melatonin is safer than and superior to placebo and is well tolerated in treating neuropathic pain.

### Design

**Figure 1:**
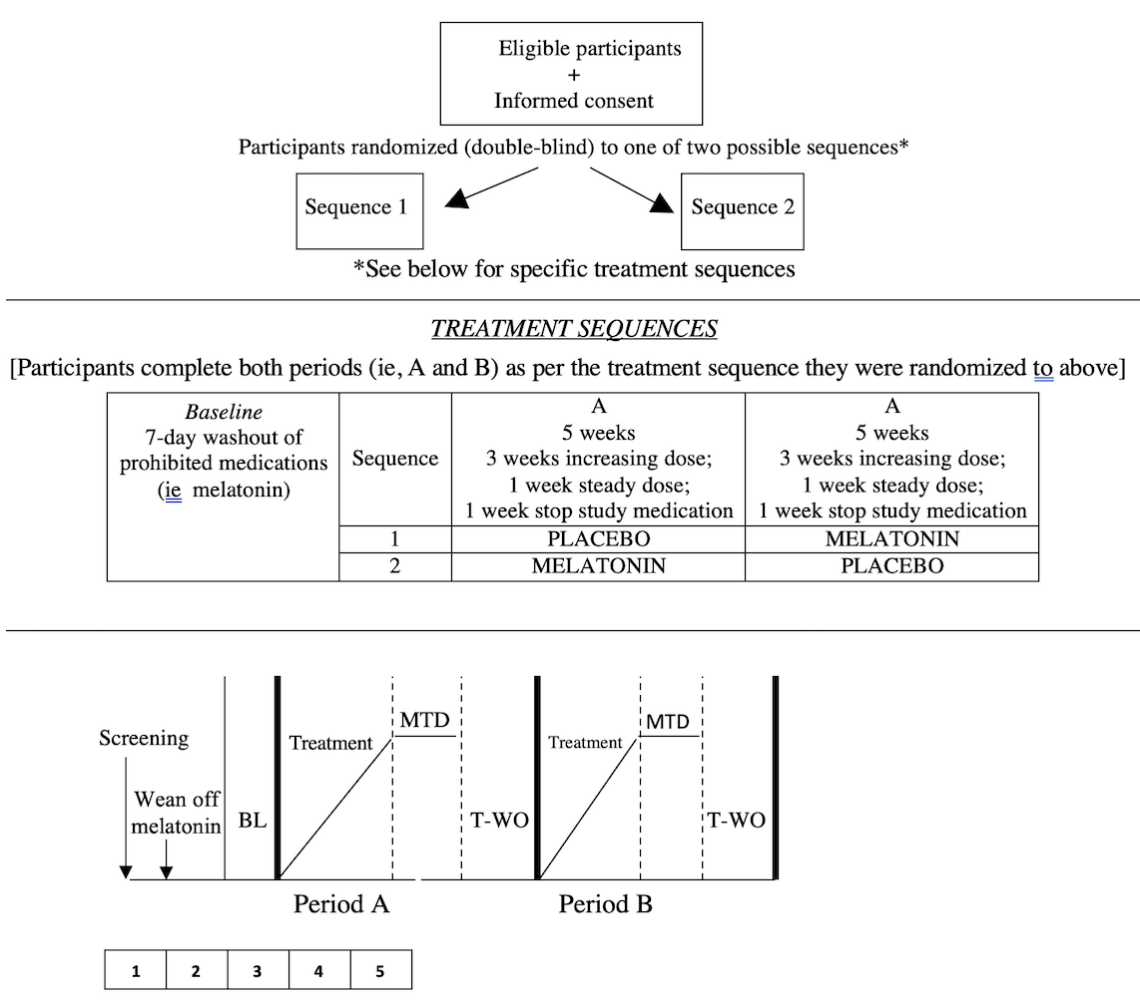
Clinical Trial Schema.

This is a double-blind, randomized, controlled, 2-period, crossover trial comparing melatonin to placebo in adults with chronic neuropathic pain (Figure 1). Both treatment periods will be 5 weeks in duration, and the entire trial will be 10 weeks long for each participant. Participants will be randomized to one of the two treatment sequences. The randomization sequence will be generated using the web-based program randomization.com (Dallal, Tufts University). Participants in sequence 1 will take active melatonin capsules during the first 4 weeks of the trial (followed by a 1-week washout period) and will subsequently take inert placebo capsules for the next 4 weeks of the trial (followed by a 1-week washout period). Participants in sequence 2 will take inert placebo capsules during the first 4 weeks of the trial (followed by a 1-week washout period) and will subsequently take active melatonin capsules for the next 4 weeks of the trial (followed by a 1-week washout period). Figure 1 shows a schematic representation of the trial design.

### Setting

Investigators work at a tertiary care health sciences center (Providence Care Hospital) in Kingston, Ontario, Canada.

### Participants

Men and women meeting the diagnostic criteria for peripheral NP will be considered for recruitment following informed consent. The inclusion criteria are as follows: (1) a score of 4 or higher on the DN4 interview—a validated questionnaire that distinguishes between neuropathic and nonneuropathic pain [24]; as indicated, investigations will be performed to confirm an NP diagnosis including, but not limited to, nerve conduction studies and electromyography; (2) daily pain (score of ≥4/10) for at least 3 months; (3) alanine transaminase level≤120% of the upper normal limit; (4) creatinine clearance of ≥60 mL/min; (5) glycosylated hemoglobin of ≤9.5%; and (6) necessary abilities, visual acuity, and language skills for questionnaire completion and telephone communication with nurses. The exclusion criteria are as follows: (1) major organ system disease; (2) cardiovascular autonomic neuropathy; (3) trigeminal neuralgia, complex regional pain syndrome, or central NP; (4) moderate to severe sedation or ataxia due to other required concomitant drugs; (5) allergy or hypersensitivity to study medications or any components in the study drug formulations or its containers; (6) seizure disorder; (7) other painful conditions that are >50% as severe as their NP; (8) a major, poorly controlled psychiatric disorder, depression or suicidal ideation, or active substance use disorder; (9) candidates who live alone and cannot assure daily contact with a friend, family member, or caregiver; (10) women of childbearing potential who will not receive a highly effective form of contraception (total abstinence, hormonal birth control methods, intrauterine devices, confirmed successful vasectomy of the partner, double barrier methods such as condom or diaphragm, etc) or a positive pregnancy test at baseline (If a study participant becomes pregnant, she must stop using study medications immediately and will be withdrawn from the study); (11) women who are breastfeeding or who plan to breastfeed; (12) regular daily administration of opioids at a dose greater than 90 mg morphine equivalents; and (13) lack of a primary care physician.

### Randomization and Blinding

We will use a balanced Latin Square crossover design [25-27] in which participants will be allocated to one of the two treatment sequences: melatonin and placebo. At the beginning of the trial, an investigational pharmacist will use a computer-generated randomization process to prepare a concealed allocation schedule to randomly assign the treatment sequences in appropriate block sizes to a consecutive series of numbers. On enrollment, each participant will be assigned to the next consecutive number, and the corresponding series of study medications will be dispensed (eg, melatonin followed by placebo or placebo followed by melatonin). Study medications will be formulated in an identical fashion across treatment periods. Treatment codes for each study participant will be generated by an investigational pharmacist and will not be disclosed to study personnel or participants until completion of the trial. Study outcome measures will be evaluated and recorded by the research study nurse who will be blinded (as will the rest of the research team) to treatment group assignments until trial completion. As an assessment of blinding to the treatment group, each participant and the study nurse will complete a blinding questionnaire at the end of each treatment period.

### Cointerventions and Rescue Medication

Any enrolled participants already taking melatonin will be weaned off during a pretrial washout of at least 7 days. Participants taking and perceiving benefit from opioids (<90 mg morphine equivalents), antidepressants (tricyclic, selective serotonin reuptake inhibitor, or serotonin-norepinephrine reuptake inhibitor), nonsteroidal anti-inflammatory drugs, or acetaminophen may continue these medications at a steady daily dose for the duration of the study. Any ongoing cognitive behavioral therapy or exercise programs perceived as beneficial will be allowed to continue only if it is certain that these will be evenly used throughout the trial. Participants will not be allowed to start new medications, cognitive-behavioral therapy, or exercise programs at any point during the study. Participants will be required to avoid any procedural pain therapies (eg, neurosurgical interventions, nerve blocks, or acupuncture) during the study. Participants will be permitted to take acetaminophen (≤8 tablets, 325 mg/tablet daily) for inadequate pain relief only during the taper and washout phases of each treatment period. Acetaminophen consumption will be recorded as a secondary outcome measure.

### Study Treatment Dosing Schedule

During each period of this trial, participants will receive 1 set of capsules (melatonin capsules, Bio-tech Pharmacal) containing 3 mg melatonin or placebo (lactose capsules). Each period will last 5 weeks, with a 4-week treatment period and a 1-week washout period. During week 1 of each treatment period, participants will take 1 capsule before bedtime. During week 2, participants will take 2 capsules before bedtime. During week 3 participants will take 3 capsules before bedtime, and during week 4, participants will take 4 capsules before bedtime. The ceiling dose for melatonin is 12 mg/day. With each increase in the dosage of study medication in the titration schedule, if mild to moderate treatment-emergent AEs (eg, sedation) are encountered, participants will be asked if they can tolerate continuing at that dose for another 2-3 days. If so, this dosage will be continued with the expectation that tolerance to side effects will occur. If side effects are severe, intolerable, or do not improve, both study medications will be decreased to the next lowest possible dose and an increase will be attempted one more time at the next scheduled dose increase. If this again results in intolerable side effects, both study drugs will be decreased back to the previous dose, which will be defined as the maximal tolerated dose (MTD) for that individual. However, during this flexible dose titration, the final dose arrived at during the MTD week (week 4 of the treatment period) could be lower than the ceiling dose of 12 mg if side effects encountered during the dose titration (eg, excessive sedation) are suspected to be due to melatonin.

### Outcome Measures

During the trial, the study nurse will contact participants by telephone at least once a week to evaluate adverse effects, assess pain intensity, and encourage compliance. Furthermore, participants will be evaluated at the clinic on 1 of the 5 weekdays of week 4 of each treatment period for vital signs and assessment of secondary outcomes. Finally, participants will be followed up by telephone 2 weeks and 3 months following the completion of the study to document any subsequent problems or adverse events. Table 1 shows the schedule of study assessments.

The primary outcome is the mean daily “average” pain intensity [28] experienced while on the MTD of melatonin or placebo during week 4 (days 22-28). This will be determined from participants’ ratings of their “average pain over the last 24 hours” completed in patient diaries every morning using a numerical rating scale from 0 to 10. Given the potential circadian variability of pain intensity [29], clinical trial methods have involved the following: (1) requiring participants to rate their pain multiple times through the day or evening [26,27], which increases participant burden and may adversely affect participant retention or missing data or (2) requiring participants to provide a retrospective rating of their “average pain over the last 24 hours” [30], which may be susceptible to recency bias. Based on our experience, we are proposing the latter approach, although both approaches have led to valid and generalizable trial results in clinical pain trials. Secondary outcomes include frequency or severity of treatment-emergent AEs; scores on the Neuropathic Pain Symptom Inventory [31], Medical Outcomes Study Sleep Scale [32], Patient Global Impression of Change [33], Brief Pain Inventory [34], Beck Depression Inventory-II [35], Beck Anxiety Inventory [36], the short-form McGill Pain Questionnaire [37], the SF-36 survey [38], and blinding questionnaires; and acetaminophen consumption. All these outcomes will be assessed at baseline and during week 4 of each treatment period, except for AEs and acetaminophen consumption, which will be assessed weekly during each treatment period.

**Table 1:** Schedule of study assessments.

Assessments	Screen	Baseline	treatment periods				3-month posttrial completion
			First 3 weeks of treatment	Fourth week of treatment	Washout		
Days per treatment period >	–14	–7	1-21	22-28	29-35		
Present pain intensity (scale 0-10; average and worst)	✓						
Concurrent medications^a^	✓	✓	✓	✓	✓	✓	
Demographics and medical history	✓						
Vital signs and weight	✓			✓			
Clinical laboratories	✓						
Adverse events^a^	✓	✓	✓	✓	✓	✓	
Drug dispensing		✓		✓			
Drug compliance and accountability				✓			
Daily pain diaries		✓	✓	✓	✓		
Maximum tolerated dose levels			✓	✓	✓		
Medical Outcomes Study Sleep Scale		✓		✓			
Other adverse effects^a^			✓	✓	✓		
Patient global impression of change			✓	✓	✓		
Brief Pain Inventory		✓		✓			
Beck Depression Inventory–II	✓	✓		✓			
Medical Outcomes Study 36­item short­form health survey		✓		✓			
Rescue acetaminophen^a^			✓	✓	✓		
Blinding questionnaire				✓			
Neuropathic Pain Symptom Inventory		✓		✓			
Pregnancy test for women of childbearing potential	✓						

^a^Evaluated during weekly participant telephone contacts with the research nurse.

### Sample Size

Statistical considerations underlying this sample size calculation are based on the null hypothesis that there is no difference in pain intensity between the study treatments and the alternative hypothesis that melatonin is different from placebo. Systematic reviews of placebo-controlled chronic pain trials consistently reveal that significant differences between treatment and placebo groups vary between 0.5 and 1.5 points, depending on the magnitude of the placebo response in any given trial [22]. Thus, based on a previous estimated within-participant variation of 2.5 from studies on NP [26], we project that a sample size of 21 participants would allow for an 80% chance of detecting (α=.05) a mean treatment group difference of 1.5 points on a 0-10 numerical rating scale. In order to have a sample size divisible by 4, we have adjusted the sample size to 24 participants. Accounting for trial dropout rates from our previous trials and for a 2-period crossover design, we expect that the recruitment of 30 enrollees for each trial will yield the aforementioned number of completers.

### Statistical Analysis

Participants who complete both treatment periods will be included in trial analyses. When data from only one period are available, sensitivity analysis including all participants will also be performed, by assuming some reasonable but extreme values for the remaining periods. Those receiving at least one dose of study drug will be included in the safety analyses. The primary outcome will be calculated as an average of pain scores as recorded in the participant pain diaries within the last 7 days (at MTD in week 4 of the treatment), if more than 50% of the information (at least 4 days) is not missing [39]. Otherwise, mean daily pain will be treated as missing data. Sensitivity analyses based on the average of all available pain scores will also be performed to confirm the results of the primary analysis. A linear mixed model with sequence, period, treatment, and the first-order carryover effects as fixed effects and participant as a random effect [39] will be used to test whether there is any treatment difference among groups and to estimate the least squares mean of the mean daily pain intensity for each treatment group, adjusting the carryover and period effects. The comparison between melatonin and placebo will be performed on the basis of the least squares means and SDs from the linear mixed model. Sensitivity analyses will be performed using a pattern-mixture model [40] based on patterns of missing data to check the robustness of results in case data may not be missing at random.

Secondary outcomes will be analyzed similarly except that (1) only 1 measurement will be analyzed in the last week for the singular measures (ie, final week questionnaires), and (2) the scoring algorithms developed for the Brief Pain Inventory, the Beck Depression Inventory-II, the Beck Anxiety Inventory, and the SF-36 will be first used to derive the subscales or domains within these instruments, and the scores on these subscales or domains will be used as response variables in the linear mixed model analysis.

## Results

This trial has been funded by the Physicians’ Services Incorporated Foundation and was registered in the International Standard Randomized Controlled Trial registry on May 4, 2022 (ISRCTN #16215617), and conditional ethics approval was obtained on May 9, 2022 (Queen’s University Health Sciences & Affiliated Teaching Hospitals Research Ethics Board protocol number ANAE-387-22).

## Discussion

NP remains a challenging condition to treat, with current recommended pharmacological therapies providing only partial relief from pain, sometimes exacerbating other symptoms [41]. To the best of our knowledge, this proposed RCT is the first to investigate the safety and efficacy of melatonin for the treatment of NP. Given the potential improvement of pain and sleep with melatonin, we expect to observe a significant difference between the melatonin and placebo groups with respect to the primary outcome of pain and the secondary outcome of sleep in patients with NP. Although there has been some previous interest in the use of melatonin for pain management, few trials have been conducted in clinical chronic pain, and most of these have involved participants with fibromyalgia [15-17] and only one trial involved participants with NP [42]. Therefore, we expect that this trial will provide much needed evidence to further guide the appropriate use of melatonin in the management of NP.

Potential threats to this trial include challenges with patient recruitment, early dropouts, noncompliance, and protocol violations. However, the planned study design as well as our experience with similar previous chronic pain trials will mitigate these threats. In particular, careful and frequent follow-up of trial participants, timely management of treatment-emergent AEs, and open communication between participants and trial personnel are, once again, expected to minimize trial dropouts and thus maximize participant retention. Furthermore, as with our past RCTs, noncompliance, protocol violations, and early dropouts will be minimized by our proposed crossover design, thorough study participant teaching, and close weekly follow-up of participants. Variations in melatonin bioavailability across trial participants may increase the overall variability in trial results; however, compared to a parallel-group trial, our proposed crossover design will reduce the impact of such variability, whereby each participant acts as their own control.

In light of the current lack of desperately needed new improved NP therapeutics, this trial is expected to provide evidence for a safer and more effective treatment for NP. The development of this proof-of-concept RCT of melatonin in NP will facilitate future confirmatory RCTs and the implementation of melatonin into practice.

## Abbreviations

AE: adverse effect
MTD: maximally tolerated dose
NP: neuropathic pain
RCT: randomized controlled trial

## Data Availability

Study data are available from the corresponding author on reasonable request.
